# Frailty, Sarcopenia, and Cognitive Risk in Senior Living Communities: Associations with Relocation Reasons and Fitness Amenity Utilization

**DOI:** 10.3390/geriatrics11040081

**Published:** 2026-07-06

**Authors:** Chiung-ju Liu, Gabriella Ulloa, Kelly Leal, Chia-Wei Fan, Pei-Shiun Chang, Inga Wang

**Affiliations:** 1 Department of Occupational Therapy, College of Public Health and Health Professions, University of Florida, Gainesville, FL 32611, USA; ulloa.g@phhp.ufl.edu (G.U.); kellyleal@ufl.edu (K.L.); 2 Department of Occupational Therapy, AdventHealth University, Orlando, FL 32803, USA; chia-wei.fan@ahu.edu; 3 School of Nursing, Indiana University, Bloomington, IN 47408, USA; pc21@iu.edu; 4 Programs in Occupational Therapy Science, Technology & Rehabilitation, School of Rehabilitation Sciences and Technology, University of Wisconsin-Milwaukee, Milwaukee, WI 53212, USA; wang52@uwm.edu

**Keywords:** aging in place, cognitive impairment, frailty, lifestyle, physical activity, retirement, sarcopenia, senior living communities

## Abstract

Background/Objectives: Senior living communities have become a popular living arrangement for older adults seeking supportive environments for aging in place. However, older adults may enter these communities with existing health vulnerabilities. This study described the prevalence of frailty, sarcopenia risk, and cognitive risk among residents and examined their associations with relocation reasons and fitness amenity utilization. Methods: A cross-sectional survey was conducted among residents aged ≥65 years who had lived in the community for at least 3 months. Survey items included demographics, health status, living history, relocation reasons, physical activity, fitness amenity use, and screening tools for frailty, sarcopenia risk, and cognitive impairment. Descriptive statistics, Mann–Whitney U tests, and Spearman rank correlations were conducted. Results: A total of 147 residents (Mean age = 80.2 years, SD = 7.1) responded to the survey. Overall, 28.5% met criteria for frailty, 19.7% screened positive for sarcopenia risk, and 21.8% for cognitive risk. Residents who reported a health-related reason for relocation showed greater frailty (U = 410, *p* < 0.001), sarcopenia risk (U = 393, *p* < 0.001), and cognitive impairment (U = 1062, *p* = 0.01). More frequent fitness amenity use was associated with lower frailty and sarcopenia risk scores (Spearman’s Rho = −0.30 and −0.40, respectively, both *p* < 0.01), but not cognitive impairment. Conclusions: A meaningful subset of senior living residents were at risk for frailty, sarcopenia, and cognitive impairment. Routine screening and interventions promoting fitness amenity use may support healthy aging in senior living communities.

## 1. Introduction

Senior living communities, which are age-restricted residential settings, have become a popular housing option for older adults seeking to age in place [1]. These communities are intentionally designed for older people who choose to voluntarily relocate, often in anticipation of changing needs later in life [2]. Senior living communities offer a spectrum of residential options. These range from independent living environments, such as active 55-plus communities that prioritize autonomy and social engagement, to continuing care retirement communities that support aging in place by offering tiered levels of progressive healthcare and daily assistance.

In the United States, occupancy rates for independent living and 55-plus communities exceeded 90% in 2025, showing their growing demand [3]. Decisions to relocate to a senior living community are often influenced by a combination of “pull” and “push” factors [4,5,6]. Pull factors attract older adults to move into these environments. These include built-in amenities like gyms and fitness programs that support an active lifestyle, ample opportunities for socialization, and supportive services such as meals and housekeeping. In contrast, push factors compel relocation; these include declining health, functional limitations, loneliness, the burden of home maintenance, or accessibility challenges within the previous home.

The decision to relocate is often complex because of the symbolic meaning of home and individuals’ emotional attachment to both their dwelling and the surrounding neighborhood [7,8]. Such attachment can intensify the tension between the desire to stay in the current home and the need to address emerging safety and support concerns. Therefore, relocation is often prompted by current or anticipated health concerns, as moves frequently coincide with periods of health decline or are made in preparation for future care needs [9,10]. Research suggests that older adults who choose to relocate tend to have more physical health problems and greater mobility or activity difficulties compared to those who remain in place [1,11,12]. Consequently, many older adults may enter senior living communities already experiencing notable health-related challenges.

Despite the growing popularity of senior living communities, studies examining the health characteristics of older adults residing in these settings remain limited. Existing studies suggest that residents experience higher prevalence rates of frailty, sarcopenia (age-related decline in muscle function and muscle mass), cognitive decline, and functional limitations when compared to non-residents counterparts [11,13,14,15]. For example, among Australian retirement village residents, approximately one-third were classified as frail and another one-third as prefrail, with one in five reporting mobility limitations [13]. In a continuing care retirement community in Florida, sarcopenia was identified in 66% of male residents and 73% of female residents—substantially higher than rates observed in nationally representative samples [14]. Similarly, among residents living in independent units within a continuing care community in the United States, the prevalence of cognitive impairment was 35% [15]. Residents across multiple U.S. communities have also reported, on average, two more limitations in activities of daily living than non-residents [11]. Collectively, these findings indicate that a substantial proportion of senior living residents are already at risk of disability.

At the same time, senior living communities are designed to support active lifestyles by providing structured environments, communal spaces, and fitness amenities. Paradoxically, however, residents’ sedentary time appears comparable to that of the general older adult population [16,17]. One study of residents of a community in the United States reported an average of 10 h per day of sedentary activities, such as reading, TV viewing, and computer use [18]. Another study of residents across 11 communities in the United States reported a similar finding of 11 h of self-reported sedentary behavior [19]. Additionally, residents with more sedentary behaviors showed poor leg muscle strength, gait speed, balance, and physical performance [19]. Conversely, residents with higher physical activity showed better physical performance [20]. These findings suggest that, despite supportive environments, many residents maintain sedentary behavior patterns.

Sedentary behavior has also been linked to cognitive decline in older adults [21]. The association is stronger among older adults who engage in less physical activity [22]. Although these relationships are well documented in community-dwelling samples, relatively little is known about how cognitive status intersects with physical activity or sedentary behavior among residents in senior living communities. Understanding these relationships is important given the high prevalence of frailty and functional vulnerability observed in this population.

In short, current evidence suggests that residents of senior living communities may enter these environments with substantial health risks, while the supportive services and amenities available may also offer opportunities to mitigate disablement. However, research examining how relocation reasons, health status, and engagement with fitness amenities relate to one another remains limited. Therefore, the primary purpose of this study was to describe the health status of residents in senior living communities, including frailty, sarcopenia, and cognitive function. A secondary purpose was to examine the associations among reasons for relocation, health status, and the utilization of fitness amenities.

## 2. Methods

### 2.1. Design and Participant Recruitment

The study was carried out in accordance with the Declaration of Helsinki of 1975, as revised in 2013. This study employed an anonymous cross-sectional survey design and was deemed exempt from Institutional Review Board review. The exempt status was approved by the Research Division of Research Operations at the University of Florida (Protocol #: ET00021075) on 24 October 2023. Participants were recruited from senior living communities located in two medium-sized cities in Florida through study flyers, community newsletters, and/or tabling events from November 2023 to November 2024.

Participants could complete the survey electronically or on paper. Electronic survey responses were collected via Qualtrics XM (Qualtrics, Provo, UT, USA), a secure online survey platform, between November 2023 and December 2024. Eligible participants were adults aged 65 years or older who had lived in a senior living community for more than 3 months. Residents living in assisted living or skilled nursing units were excluded from the study. Potential participants were informed of the study’s purpose and the anonymity of their responses prior to completing the screening questions. Completion of the survey was considered to imply consent to participate in the study.

### 2.2. Survey Description

The survey consisted of multiple-choice, select-all-that-apply, fill-in, and dichotomous questions. Survey domains covered demographics and basic information (6 questions); general health status (2 questions); frailty (15 questions); sarcopenia (5 questions); cognitive function (8 questions); living history and reasons for relocation (4 questions); physical activity (10 questions); and the availability and use of fitness amenities within the community (5 questions).

Frailty was measured using the Tilburg Frailty Indicator (TFI) [23]. It measures frailty across physical, psychological, and social domains and includes subscales for physical frailty (8 questions), psychological frailty (4 questions), and social frailty (3 questions), with a total score ranging from 0 to 15. A score of 5 or higher indicates frailty.

Sarcopenia risk was assessed using the Strength, Assistance in Walking, Rise from a Chair, Climb Stairs, and Falls questionnaire (SARC-F) [24,25]. The SARC-F consists of five functional components, each scored from 0 to 2, yielding a total score ranging from 0 to 10. A score of 4 or higher indicates risk of sarcopenia.

Cognitive function was assessed using the Ascertain Dementia 8-Item Informant Questionnaire (AD8) [26,27]. The AD8 is a brief screening tool designed to differentiate normal aging from dementia-related cognitive decline by assessing perceived changes in cognition over the past several years. Prior research has shown adequate agreement between self-reported and informant-based scores [28]. Although the questionnaire is preferably completed by an informant, it may be administered as a self-rating tool, particularly when an informant is unavailable. Total scores range from 0 to 8. A score of 2 or higher suggests cognitive risk.

Living history included years of residence and the type of community (e.g., active living community or continuing care retirement community). Regarding reasons for relocation, residents were asked to select up to three major push and pull factors reported by a scoping review [6].

Physical activity was measured by the International Physical Activity Questionnaire-Short Form (IPAQ-SF) [29]. The IPAQ-SF consisted of 6 questions designed to estimate the intensity, frequency, and duration of physical activity, including walking, moderate-intensity, and vigorous-intensity activities, performed in the past 7 days. Based on reported activity intensity, frequency, and duration, each resident’s physical activity level was categorized as high, moderate, or inactive [30]. The IPAQ-SF also included one question assessing time spent sitting. In addition, the survey included supplementary questions to capture residents’ physical activity. These included three dichotomous items assessing engagement in muscle-strengthening exercises and light or heavy housework in the past 7 days, as well as four items assessing the types and use of fitness amenities within the community. Three questions asked respondents to report on the fitness amenities in their communities (e.g., gym facilities, group exercise programs, personal training, outdoor programs), the types of amenities they usually used (e.g., exercise equipment, group fitness classes, personal training, swimming pool, golf course, tennis court), and the frequency of use over the past 7 days (did not use, 1–2 days, 3–5 days, almost every day). A final question assessed the average amount of time spent using these fitness amenities each day.

### 2.3. Data Analysis

Survey responses were summarized using descriptive statistics, with categorical variables reported as frequencies and percentages and continuous variables as means and standard deviations. Descriptive results were organized by respondent characteristics, relocation reasons, physical activity and fitness amenities, and indicators of frailty, sarcopenia, and cognitive function.

Group differences in frailty (TFI), sarcopenia risk (SARC-F), and cognitive function (AD8) by relocation reason (health-related vs. not) were examined using the Mann–Whitney *U* test. Correlations between measures of TFI, SARC-F, and AD8 and the frequency of fitness amenity use were assessed using Spearman’s rank correlation coefficients (*ρ*). All tests were two-sided with α = 0.05. Nonparametric statistics were used because the measures described above (e.g., TFI, SARC-F, and AD8) yield ordinal data, for which such methods are more appropriate [31]. A formal power analysis was not conducted because this study was designed as a descriptive survey rather than a priori hypothesis-testing research.

## 3. Results

A total of 147 residents with a mean age of 80.2 years (SD = 7.1) completed the survey. The majority were female and White. The mean years of education was 16.9 (SD = 2.8). About 58% of respondents lived alone, and 40% lived with a spouse or partner. Respondents lived in their current community for a mean of 6.8 years (SD = 6.3). Nearly half lived in an active living community (51.1%), followed by a continuing care retirement community (43.6%). The mean number of self-reported chronic conditions was two (SD = 1.6). Most reported being in good health and felt about the same compared to one year ago. Respondent characteristics and general health status are summarized in Table 1.

### 3.1. Push and Pull Factors for Relocation

Table 2 summarizes the top selections of push and pull factors. The reasons respondents moved away from their prior community residences were more diverse. The top reasons were the desire to plan ahead while they were still able and home maintenance responsibilities. The plan to change lifestyle, declining health, fear of burdening others, and feeling isolated were also common. On the other hand, the reasons for moving to a senior living community were more convergent. The top selected reasons included the availability of community amenities and programs, anticipation of future long-term care needs with the option to move into units with higher levels of support, social engagement opportunities, and the desire to maintain a preferred lifestyle.

### 3.2. Physical Activity and Fitness Amenity Use

Based on the IPAQ-SF, 76 participants (51.7%) reported engaging in vigorous activities; 90 (61.2%) reported engaging in moderate activities, and 124 (84.4%) reported walking for at least 10 min at a time in the last 7 days. Of 139 residents with sufficient information to estimate the physical activity level, 39 (26.5%) were categorized as high, 69 (46.9%) as moderate, and 31 (21.1%) as inactive.

Regarding sitting time, only 90 residents provided estimates in minutes. Among these respondents, 60 (66.7%) reported sitting for fewer than 5 h per day, 23 (25.6%) for 5 to 9 h, and 7 (7.8%) for 10 or more hours. Additionally, 91 residents (61.9%) reported performing exercises specifically to increase muscle strength and endurance during the past 7 days, 136 (92.5%) reported performing light housework, and 47 (32.0%) reported performing heavy housework.

Table 3 shows the community fitness amenities reported and those frequently used by respondents. Most communities provided fitness or physical activity programs. Respondents often used exercise machines or equipment, as well as group fitness classes. Nearly one-third of respondents reported using these fitness amenities on 3 to 5 days during the past 7 days. Most residents reported using the amenities for 30 to 120 min per day.

### 3.3. Frailty, Sarcopenia, and Cognitive Function

The TFI score ranged from 0 to 10, with a mean of 3.4 (SD = 2.6). Forty-two respondents (28.5%) had a TFI score of 5 or higher, indicating frailty. The SARC-F score ranged from 0 to 8, with a mean of 2.0 (SD = 2.2). Twenty-nine respondents (19.7%) had a SARC-F score of 4 or higher, indicating a risk of sarcopenia. The AD8 score ranged from 0 to 8, with a mean of 1.1 (SD = 1.7). Thirty-two respondents (21.8%) had a score of 2 or more, indicating cognitive risk.

Table 4 shows comparisons of frailty, sarcopenia, and cognitive function between respondents whose relocation was health-related (i.e., the push factor of declining health) and those whose relocation was not. Respondents who reported that the relocation reason was health-related showed greater frailty, sarcopenia risk, and cognitive impairment, indicated by higher scores on TFI (U = 410, *p* < 0.001), SARC-F (U = 393, *p* < 0.001), and AD8 (U = 1062, *p* = 0.01). Additionally, the frequency of fitness amenity use was significantly correlated with frailty and sarcopenia risk. More frequent use was moderately correlated with lower TFI scores (Spearman’s *ρ* = −0.30, *p* < 0.001) and SARC-F scores (Spearman’s *ρ* = −0.40, *p* < 0.001). However, the correlation between fitness amenity use and cognitive function, as measured by the AD8, was weak and not statistically significant (Spearman’s *ρ* = −0.10, *p* = 0.16).

## 4. Discussion

The purpose of this study was twofold: to describe the health status of older adults residing in senior living communities and to examine associations among reasons for relocation, health status, and the use of fitness amenities. The majority of study respondents reported good health and an active lifestyle, consistent with the main relocation reasons of early planning and the attractions of community fitness amenities and programs. However, despite over half of the respondents engaging in physical activities in the past 7 days, approximately 20% to 30% were at risk of frailty, sarcopenia, and cognitive impairment. In particular, residents who relocated for health-related reasons exhibited higher levels of frailty, sarcopenia risk, and cognitive risk than those who did not, suggesting that health status among residents might differ at the time of transition. In addition, more frequent use of fitness amenities was associated with lower levels of frailty and sarcopenia risk, but not with cognitive risk. Given the cross-sectional design, the direction of these associations cannot be determined. 

The mean age of the study respondents was 80 years, which is slightly younger than that of participants in several prior studies of senior living communities [14,15,19,20]. Based on the reported average length of residence of 7 years, most residents were likely to have moved into the community in their early 70s, reflecting their reported top push factor for relocation, the desire to plan ahead while they still can.

The late-life migration may be interpreted through the environmental press model [32]. The model posits that optimal functioning reflects a balance between personal competence and environmental press (demand), and that older adults adapt their environments to maintain that balance. As older adults experience changes in physical, cognitive, or social competence, the press of the prior home may increase, making tasks such as home maintenance and heavy housework more challenging [33]. Within this model, many respondents in our study proactively relocated to a senior living community early in late life to reduce environmental press and access resources that support their competencies. This study showed that respondents who relocated because of a health-related reason tended to have frailty, sarcopenia, and cognitive risk. This finding aligns with Shinan-Altman et al. (2020), who reported that among U.S. older adults, relocation to a continuing care retirement community was associated with more positive attitudes toward the community, higher well-being, but poorer subjective health [11].

The study findings added to the limited evidence that residents of senior living communities are not uniformly robust. In this study, 29% of respondents were classified as frail, which is somewhat lower than the 34% reported by Cobden et al. (2022) among Australian retirement village residents [13]. Nonetheless, both studies identify a subgroup of residents with increased vulnerability. Similarly, 20% of our sample screened positive for sarcopenia, which is lower than the prevalence of low muscle mass reported by Hunt et al. (2018) in a Florida continuing care retirement community (66% in men and 73% in women) [14]. Additionally, 22% of respondents screened at risk for cognitive impairment, which is much lower than the 35% reported by Van Patten et al. (2022) in a California continuing care retirement community [15]. Differences in prevalence estimates across studies may be partly attributable to variation in measurement approaches, for example, the use of a brief self-report functional screening tool (e.g., SARC-F, AD8) versus direct quantification of muscle mass and global cognitive function. Furthermore, the current study focused on residents in independent living units and excluded those in assisted living, who may have a poorer health status. Regardless of these differences in prevalence rates, the findings highlight the value of routine screening to identify at-risk residents in senior living communities.

The results also suggest that respondents were relatively active, with more than 50% engaging in moderate to vigorous physical activity in the past 7 days. Most performed strengthening or endurance exercises. Additionally, the respondents reported fewer sitting hours compared with prior research [18]. Consistent with this, the use of fitness amenities was frequent, typically exceeding two days per week and more than 30 min per session. The active lifestyle corroborates the inverse correlations identified between fitness-amenity use and frailty or sarcopenia risk. However, the absence of a similar association with cognitive risk remains unclear. While physical activity is known to protect against cognitive decline [34], other unmeasured factors, such as social networks, may moderate the correlation [35]. Particularly, over half of the respondents in this study lived alone. Prior research suggests that social engagement may be particularly important in this context; for example, a longitudinal study found that participation in formal social activities during the first year of residence in independent living was associated with less decline in quality of life over five years [36]. Similarly, another large cohort study suggested that social participation plays a role comparable to physical activity in maintaining cognitive health in older adults [37]. Some residents might not be physically active, but were socially active. Given that many senior living communities offer organized activities, communal dining, and group outings, these environments may promote social connectedness and cognitive stimulation, potentially influencing cognitive outcomes.

## 5. Limitations

Study findings should be interpreted with caution, as causal relationships cannot be established due to the cross-sectional study design. Additionally, the reliance on univariate analyses limits the ability to account for potential confounding variables that may influence the observed associations. The relatively modest sample size constrained the feasibility of conducting multivariate analyses. Finally, the use of a non-probability sampling approach may have introduced selection bias, resulting in a sample that is younger, more active, and healthier than the broader target population, potentially limiting the generalizability of the findings.

## 6. Conclusions

The current study identified a subset of residents of senior living communities at elevated risk for frailty, sarcopenia, and cognitive impairment. These risks appear to be associated with health-related reasons for relocation. Use of community fitness amenities was associated with lower risk of frailty and sarcopenia, but not with cognitive risk.

Despite some study limitations, the study underscores the importance of routine screening to identify high-risk residents in senior living communities. Future research is warranted to examine the effects of supportive services and amenities on mitigating cognitive and physical decline among this population using a larger longitudinal study design.

## Figures and Tables

**Table 1:** Characteristics of Survey Respondents (n = 147).

Characteristics	n	Percent
Age in years		
65–74	33	22.5%
75–84	78	53.1%
85 and above	34	23.1%
Missing	2	1.4%
Gender		
Female	105	71.4%
Missing	2	1.4%
Race		
Asian, Asian American	2	1.4%
Black or African American	1	0.7%
White	140	95.2%
Others or prefer not to answer	3	2.1%
Missing	1	0.7%
Ethnicity		
Hispanic or Latino	1	0.7%
Non-Hispanic or Latino	134	91.2%
Prefer not to answer	3	2.0%
Missing	9	6.1%
Years of education		
Less than 12 (less than a high school degree)	1	0.7%
12 (a high school diploma or GED)	14	9.6%
13–16 (some college, associate degree, or bachelor’s degree)	49	33.3%
More than 16 (graduate or professional degree)	79	53.7%
Missing	2	1.4%
Living arrangement		
Living alone	85	57.8%
Living with a spouse	56	38.1%
Living with a partner	3	2.0%
Living with others (e.g., a friend)	2	1.4%
Missing	1	0.7%
Years living in the community		
1 year or less than 1 year	17	11.6%
2 to 5 years	64	43.5%
6 to 10 years	35	23.8%
Above 10 years	29	19.7%
Missing	2	1.4%
Number of chronic conditions		
2 or less	96	65.3%
3 to 5	36	24.5%
6 or more	4	2.7%
Missing	11	7.5%
Self-reported general health		
Excellent	35	23.8%
Very good	59	40.1%
Good	38	25.9%
Fair	9	6.1%
Poor	6	4.1%
Self-reported health status compared to one year ago		
Much better	15	10.2%
Somewhat better	14	9.5%
About the same	91	61.9%
Somewhat worse	23	15.7%
Much worse	4	2.7%

**Table 2:** Summary of Push and Pull Factors for Relocation.

Factor Options Provided in the Survey	n	Percent
Push Factors		
Desire to plan ahead while still able	69	46.9%
Home maintenance of prior residency	63	42.9%
Change in lifestyle	35	23.8%
Declining health	25	17.0%
Fear of burdening others	25	17.0%
Felt socially isolated	24	16.3%
Heavy housework in prior residency	17	11.6%
Declining health of others	15	10.2%
Lack of services in prior residency	13	8.8%
Needed assistance and no one was available to help	7	4.8%
Reduction in income	6	4.1%
Pull Factors		
Community amenities and programs	114	77.6%
Prospect of receiving long-term care in the future	50	34.0%
Desire to maintain a preferred lifestyle	46	31.3%
Social engagement opportunities	46	31.3%
Being with people of similar ages	32	21.8%
Offer household maintenance services	30	20.4%
To be close to family, friends, or neighbors	29	19.7%
Affordability	27	18.4%

**Table 3:** Community Fitness Amenities and Use.

Variables	n	Percent
Community fitness amenities		
Group exercise programs	60	40.8%
Gym or fitness center	59	40.1%
Outdoor physical activity programs (e.g., golf courses and tennis courts)	44	29.9%
Personal training	32	21.8%
Frequently used fitness amenities		
Exercise machines or equipment	73	49.7%
Group fitness classes	70	47.6%
Swimming pool	46	31.3%
Personal training	17	11.6%
Golf course	9	6.1%
Tennis court	1	0.7%
Do not use any	6	4.1%
Frequency of fitness amenity use over the past 7 days		
1–2 days	27	18.4%
3–5 days	47	32.0%
Almost every day	27	18.4%
Did not use	38	25.9%
Missing	8	5.4%
Minutes of fitness amenity use per day		
Less than 30 min	9	6.1%
30 to <60 min	42	28.6%
60 to <120 min	34	23.1%
120 min or more	5	3.4%
Missing	50	34.0%

**Table 4:** Comparisons of Frailty, Sarcopenia, and Cognitive Function Between Respondents with a Health-related Relocation Reason and Those Without.

Measure	Respondents with a Health-Related Relocation Reason Mean (SD)	Respondents Without a Health-Related Relocation Reason Mean (SD)	Mann–Whitney U	*p* Value
Tilburg Frailty Indicator (TFI)	6.2 (2.1)	2.8 (2.3)	U = 410	*p* < 0.001
Strength, assistance in walking, rise from a chair, climb stairs, and falls (SARC-F)	4.5 (2.3)	1.4 (1.6)	U = 393	*p* < 0.001
Ascertain Dementia 8-item Informant Questionnaire (AD8)	1.9 (2.2)	0.9 (1.4)	U = 1062	*p* = 0.01

## Data Availability

The raw data supporting the conclusions of this article will be made available by the authors on request.
